# Using Collective Impact for intersectoral action in rural Northern Ontario: Two case studies

**DOI:** 10.17269/s41997-025-01137-y

**Published:** 2025-12-29

**Authors:** Amanda Mongeon, Erin Cowan, Walter Humeniuk, Shujian Liu, Leith Deacon

**Affiliations:** 1 Northeastern Public Health, Temiskaming Shores, ON Canada; 2 University of Guelph, Guelph, ON Canada

**Keywords:** Intersectoral collaboration, Rural health, Public health, Community health planning, Health equity, Collaboration intersectorielle, Santé en zone rurale, Santé publique, Planification de la santé communautaire, Équité en santé

## Abstract

**Setting:**

Timiskaming District in Northern Ontario has a population of 32,394 people across 24 municipalities, two unincorporated areas, and four First Nations. During the time of these case studies, public health services were provided by the Timiskaming Health Unit, one of 34 local public health agencies in Ontario.

**Intervention:**

To address local public health priorities in this rural region, the Timiskaming Health Unit implemented the Collective Impact framework, establishing governance structures for two initiatives: the Timiskaming Community Safety and Wellbeing Plan (CSWB) and Timiskaming Drug and Alcohol Strategy (TDAS). Acting as the backbone organization, the Health Unit facilitated a common agenda, shared progress measures, and coordinated mutually reinforcing activities.

**Outcomes:**

The 2023 CSWB Plan, co-funded by all 24 municipalities, established a steering committee and three working groups to address safety and well-being goals. TDAS, launched in 2022, engages over 20 organizations and community members through a steering committee and four working groups. Deliverables include public events, navigational resources, social marketing campaigns, capacity building, new health infrastructure, improved collaboration, and advocacy for healthy public policy.

**Implications:**

These initiatives demonstrate how local public health units can use the Collective Impact framework to address complex rural public health challenges. By integrating a continuous learning approach, implementation can integrate knowledge to foster collaboration that leads to community engagement and policy change. However, sustainable funding is critical for supporting collaborative governance and mitigating challenges like limited rural data availability.

## Résumé

**Cadre:**

Le district de Timiskaming, dans le Nord de l’Ontario, compte 32 394 habitants répartis entre 24 municipalités, deux secteurs non constitués en municipalités et quatre Premières Nations. Pendant la période de nos études de cas, les services de santé publique étaient fournis par les Services de santé du Timiskaming, l’un des 34 bureaux de santé locaux de l’Ontario.

**Intervention:**

Pour aborder les besoins prioritaires en santé publique de cette région rurale, les Services de santé du Timiskaming ont mis en œuvre le modèle d’impact collectif en établissant les structures de gouvernance de deux initiatives: le Plan de sécurité et de bien-être communautaires (SBEC) du district de Timiskaming et la Stratégie contre les drogues et l’alcool du Timiskaming (SDAT). Agissant à titre de réseau fédérateur, les Services de santé ont facilité un programme commun, partagé des indicateurs de progrès et coordonné des activités synergiques.

**Résultats:**

Dans le cadre du Plan de SBEC 2023, cofinancé par les 24 municipalités du district, un comité directeur et trois groupes de travail ont été formés pour aborder des objectifs de sécurité et de bien-être. La SDAT, lancée en 2022, implique plus de 20 organismes, ainsi que les membres de la communauté, au sein d’un comité directeur et de quatre groupes de travail. Les livrables incluent des manifestations publiques, des ressources de navigation, des campagnes de marketing social, le renforcement des capacités, de nouvelles infrastructures de santé, une collaboration améliorée et la promulgation de politiques publiques favorables à la santé.

**Implications:**

Ces initiatives montrent que les bureaux de santé locaux peuvent se servir du modèle d’impact collectif pour aborder des problèmes de santé publique complexes en milieu rural. En adoptant une démarche d’apprentissage continu, la mise en œuvre d’initiatives peut intégrer les connaissances pour favoriser une collaboration qui mène à la mobilisation communautaire et à la réorientation des politiques. Un financement durable est toutefois essentiel pour appuyer la gouvernance concertée et aplanir les difficultés comme le manque de données rurales.

Health outcomes in Northeastern Ontario’s Timiskaming Health Unit Area are shaped by complex social and structural factors. The district spans 14,146 km^2^ with a population density of 2.3 people per km^2^ (Statistics Canada, 2023). Indicators for health, education, employment, and income are worse in Timiskaming compared to the provincial average (Statistics Canada, 2022, 2023). For example, life expectancy is nearly 4 years less than that of the average Ontarian (Statistics Canada, 2019), and chronic health conditions such as chronic obstructive pulmonary disease (COPD), diabetes, and high blood pressure are more prevalent (Statistics Canada, 2022). Educational attainment is also lower, with fewer high school and university graduates per capita than Ontario (Statistics Canada, 2023). Food security is a significant challenge; food deserts exist in several areas and 22.6% of households are food insecure (Statistics Canada, 2023). Household income in Timiskaming is also statistically lower than the provincial average (Statistics Canada, 2023), and in 2021, 10.7% of private dwellings required major repairs (Statistics Canada, 2023).

## Timiskaming Health Unit

The Timiskaming Health Unit (THU)^1^ is one of 34 boards of health in Ontario tasked with promoting population health. Ontario’s public health programs are organized into four domains: social determinants of health, healthy behaviours, healthy communities, and population health assessment. Guided by the principles of need, impact, capacity and partnership, collaboration, and engagement (Ontario Ministry of Health, 2021), THU leverages its deep understanding of local contexts, strong partnerships, and experience addressing complex public health issues to support local implementation of the Collective Impact framework.

## Collective Impact for complex rural social problems

Addressing complex health issues requires intersectoral collaboration and transdisciplinary approaches targeting root causes (Gadsby & Wilding, 2024; Mulligan, 2022). This is particularly true in rural communities, where historical, geographic, and social factors distinguish them from urban areas and from one another (Markey et al., 2020). While collaborative, place-based action is useful in all communities, it is especially well suited to rural ones.

Effectively addressing complex social issues also requires interventions targeting root causes. For example, promoting substance use health necessitates investments across the social-ecological spectrum (Canadian Public Health Association, 2024), such as supportive housing, positive parenting, education, community programming, and trauma-informed health services. Failure to account for this complexity results in reactive, narrowly focused interventions, forcing trade-offs among critical issues that could instead be jointly addressed through coordinated efforts.

Rural communities have distinct characteristics. Complex issues manifest differently due to distinct social, economic, and environmental factors (Kevany & Fromstein, 2020), necessitating tailored governance approaches (Mongeon et al., 2023). For example, rural areas often require greater per-person investment due to low population density (Harris et al., 2016). Additionally, variations in local governance structures (e.g. single-tier vs. two-tier municipalities) and the diverse knowledge and experience of local actors lead to unique, locally governed solutions.

The Collective Impact (CI) model, introduced in 2011 (Weaver & Cabaj, 2018), facilitates collaboration to address complex social problems. While the model has evolved over time, at its core are five parameters: a common agenda, shared measurement, mutually reinforcing activities, continuous communication, and backbone support. The framework also specifies three preconditions for Collective Impact: financial resources for 2–3 years, influential champions, and a sense of urgency around the issues being addressed (Weaver & Cabaj, 2018). Despite its widespread adoption, critics highlight gaps such as insufficient community engagement and a failure to address systemic inequities (Ennis & Tofa, 2019).

Weaver and Cabaj (2018) describe the evolution of the CI framework into “Collective Impact 3.0”, updating the framework to address limitations identified during its first 5 years of implementation. This evolution, summarized in Table 1, includes greater emphasis on community engagement to support place-based interventions, upstream prevention, systems change, and an integrated philosophy of evaluation and learning. This updated framework also shifts from a managerial, institution-led approach to one of movement building, promoting collaboration among diverse actors to build on community assets and drive systemic change (Weaver & Cabaj, 2018).

**Table 1:** Evolution of Collective Impact to Collective Impact 3.0 (Weaver & Cabaj, 2018)

Five conditions for Collective Impact	Updated conditions in Collective Impact 3.0
Common agenda	Community aspiration
Shared measurement	Strategic learning
Mutually reinforcing activities	High leverage activities
Continuous communication	Inclusive community engagement
Backbone	Containers for change

## Purpose of the article

This article presents THU’s adaptive use of the CI framework as a means for local public health to meet its mandate in a rural setting through two collaborative initiatives focused on community well-being and safety. It explores the evolution from CI to CI 3.0, identifying areas where updated factors have already been integrated and opportunities for others to be put in place. Other local public health agencies may find these examples of CI implementation useful in informing their work in co-creation and collaborative governance for health and well-being.

## Case studies

### Case study A: Timiskaming community safety and wellbeing (CSWB) plan

In 2022, Ontario mandated municipalities to develop Community Safety and Wellbeing (CSWB) Plans. Timiskaming’s 24 municipalities collectively adopted a plan identifying six priorities: health and well-being, housing, employment and economy, poverty, community safety, and environment and sustainability (District of Timiskaming Social Services Advisory Board, 2022). Implementation began in 2023, supported by a Steering Committee of representatives from municipal, health, and social service sectors. Working groups were established for three initial priorities—health and well-being, housing, and community safety—bringing together diverse partners from the health, social services, justice, municipal, education, private, and non-profit sectors. These groups focus on collaborative interventions for complex local issues, specifically focusing on health equity, anti-racism, advocacy, and respect for Indigenous culture, history, and tradition as articulated in both the plan and the Steering Committee’s Terms of Reference (District of Timiskaming Social Services Administrative Board, 2022).

#### Accomplishments

Achievements include a housing inventory, local adaptation of the 211 social service directory, expanded outreach to older adults, creation of a justice navigation map, and establishment of a local Situation Table for rapid incident response. Collaboration with the local community foundation is leading to the production of the community Vital Signs report, while efforts to bridge the CSWB with Ontario Health Team initiatives have fostered connections between municipal and community actors.

#### Challenges and sustainability

The initiative faces sustainability challenges due to reliance on a single full-time coordinator whose role is currently cost-shared by municipalities. While project-specific funding is accessible through sources like the United Way, the local community foundation, and government programs, they are resource-intensive to access, and sustainable funding for the coordinator remains a critical challenge. Small rural tax bases and increasing municipal responsibilities exacerbate funding pressures, threatening the long-term viability of the initiative. While there is an existing indicator framework explicitly developed for CSWB implementation in Canada (Canadian Municipal Network on Crime Prevention, 2020), access to local meaningful data to evaluate success is limited due to infrequent occurrences and small sample sizes in rural communities.

### Case study B: Timiskaming drug and alcohol strategy (TDAS)

The opioid crisis, along with normalized alcohol use and persistent tobacco consumption, has had a profound impact on Northern Ontario, resulting in high human and system costs. During the COVID-19 pandemic, the Timiskaming area experienced alarming rates of substance-related harms. In 2020, emergency department visits (239.4 per 100,000) and hospitalizations (104.6 per 100,000) for cannabis-related harms were significantly higher than provincial rates (113.0 per 100,000 and 49.3 per 100,000 respectively) (Ontario Agency for Health Protection and Promotion (Public Health Ontario), 2024a), as were alcohol-attributable hospitalizations (294.0 per 100,000 vs 203.1 per 100,000) (Ontario Agency for Health Protection and Promotion (Public Health Ontario), 2024b). Most alarming, the district recorded its highest-ever number of opioid-related deaths with seven confirmed deaths in 2020 (Ontario Agency for Health Protection and Promotion (Public Health Ontario), 2024c).

A structured, collaborative framework action plan on substance use is commonly known as a drug strategy, and the four-pillar approach focuses on prevention, treatment, harm reduction, and enforcement. While federal strategies like the Canadian Drugs and Substances Strategy implement this approach to set national standards and enable legislative changes (Health Canada, 2024), local strategies offer the flexibility to tailor interventions to unique community needs. For example, Vancouver’s strategy, informed by public consultation, has made several pivots to innovate harm reduction by introducing North America’s first supervised consumption site (MacPherson et al., 2006), while Waterloo Region’s enforcement pillar directs attention to justice initiatives, such as Drug Treatment Courts which direct focus to treatment and rehabilitation from punitive measures (Waterloo Region Integrated Drugs Strategy, 2018).

#### Development of TDAS

In 2021, with funding from the Public Health Agency of Canada and guidance from the Drug Strategy Network of Ontario, Timiskaming partners began working on the TDAS. Following community consultation, the final TDAS was released (Timiskaming Health Unit, 2022). Using the four-pillar approach, TDAS aims to create Collective Impact by aligning the goals and metrics of local community partners in relevant sectors. Guiding principles emphasize equity, trauma and violence-informed approaches, and reconciliation awareness in all aspects of TDAS. THU hosts a paid coordinator who facilitates collaboration, ensures connection among the pillars, leads communication, and supports planning and implementation.

#### Accomplishments

With THU as the backbone organization, TDAS has fostered partnerships with over 20 organizations, including health and social services, Indigenous communities, municipal partners, law enforcement, school boards, and non-profits who share staff capacity, in-kind resources, data, communications, and advocacy efforts.

Achievements include adopting the Icelandic Prevention Model for youth substance use (well described in Sigfusdottir et al., 2019) and local, provincial, and federal advocacy on substance use policy. A Substance Use Referral Pathway Tool was developed to improve system navigation; initially an online directory, it evolved into a clinic-based referral tool for frontline staff, now widely used by pharmacies, walk-in clinics, and peer supporters. A local community of practice has delivered training related to trauma- and violence-informed care, structural stigma, and harm reduction. In addition, through collaborations with organizations outside the district, TDAS played a key role in expanding local harm reduction services. Notably, the partnership with the AIDS Committee of North Bay and Area led to the launch of a peer outreach program, and with support from St. Michael’s Hospital, the installation of the Our Health Box dispensing machine.

### Challenges

While one-time funds from various sources, including the Public Health Agency of Canada, Timiskaming Health Unit, and Northern Ontario Heritage Fund Corporation, have supported the TDAS coordinator position, the lack of stable funding to sustain the position and delayed access to health outcome data such as substance use rates present challenges. Progress and efficacy of individual initiatives are measured and used as proxies for the overall success of the strategy (e.g.Humeniuk, 2024; Planet Youth Timiskaming, 2023).

## Discussion

### Common agenda → community aspiration

Both TDAS and CSWB have developed high-level plans with varying degrees of community and organizational input, serving as a common agenda for collaborative initiatives. However, by building on efforts to engage more intentionally and meaningfully with the broader community, both initiatives have the potential to cultivate a more aspirational vision—one that inspires community members to actively contribute.

Planet Youth Timiskaming (PYT), an intervention incubated by TDAS, demonstrates this well. While PYT’s raison d’etre is youth substance use prevention, its broader, aspirational goals—increasing the constructive use of free time among youth, fostering a sense of belonging and connection to the community, and raising awareness of protective factors to positive youth development—have attracted widespread participation. Educators, faith leaders, non-profit organizations, parents, local governments, and others have engaged enthusiastically with these goals.

When TDAS and CSWB move forward with renewing their plans and setting updated objectives, adopting a more aspirational approach that harnesses the energy and commitment of a diverse array of community actors could lead to even more meaningful and significant results.

### Shared measurement → strategic learning

Both TDAS and CSWB leverage local public health expertise in effective practice, including access to research evidence. As their Terms of Reference come up for renewal, both initiatives could benefit from exploring the shift to strategic learning within the CI 3.0 framework. However, rural communities in Canada face significant challenges in health data availability, including issues related to timing, quality, and local relevance (Morris et al., 2021). These challenges make meaningful outcome measurement particularly difficult, hindering the ability of initiatives like TDAS and CSWB to monitor and report on their progress effectively.

Both initiatives have used proxy measures to report on the cumulative progress of interventions that are independently evaluated. While this approach helps mitigate some of the challenges, it is not without limitations.

### Backbone → containers for change

The Collective Impact model has allowed THU to facilitate functional governance structures, maintain multiple working groups with individual workplans, and unite diverse community partners under a shared agenda. THU also leads a measurement strategy for each initiative to ensure collective alignment on priorities and progress. While funding was secured in both initiatives for the initial years of implementation, these distinct initiatives require resources for co-created interventions and governance, and grapple with resource limitations typical of rural areas. Unlike larger municipalities, which often fund such initiatives, rural areas face expanding responsibilities while operating with limited tax bases and staffing (Gibson & Dale, 2022). These challenges include maintaining infrastructure under evolving government mandates, addressing housing needs amid competing land demands, adapting to shifting economies, and navigating governance and financing systems that are often misaligned with rural realities (Gibson & Dale, 2022). Additional pressures, such as reliable broadband access, strengthening relationships with Indigenous communities, and supporting aging populations, further strain small local governments (Rural Ontario Institute, 2019). While there is a strong willingness to collaborate, funding for coordination and governance, as well as for co-created or collaborative interventions, remains scarce.

### Continuous communication → inclusive community engagement

CSWB and TDAS prioritize ongoing communication within their governance structures and with the broader public. TDAS, from its inception, has emphasized meaningful community engagement through the establishment of a People with Lived and Living Experience Advisory Committee. Members of this committee are compensated for their time and contribute by identifying community needs, advising on plans and their implementation, and pursuing self-determined educational opportunities.

In contrast, while similar community engagement was discussed during the early stages of CSWB’s implementation, such a mechanism has not yet been established. Developing a robust approach to community engagement remains an area for future development.

### Mutually reinforcing activities → high leverage activities

Locally driven interventions addressing risk and protective factors, like the CSWB Plan and TDAS, provide substantial return on investment (Health Promotion Ontario, 2023). These two initiatives have helped build social capital among collaborators and promote institutional support for community change by engaging with people with lived experience. They have also led to collective advocacy for upstream policy change that individual member organizations may not do on their own, due to political sensitivities or capacity limitations.

Both CSWB and TDAS have led to projects that might not otherwise have been realized or would have been executed with less engagement and lower quality. For example, smaller, task-specific groups within these initiatives have successfully completed projects like the TDAS Substance Use Referral Flow Chart and the CSWB’s South Timiskaming Situation Table. Initially drafted by public health, collaborative communications campaigns were significantly enhanced through partner input. For both initiatives, steering committees provide direction and accountability, but progress comes from their working groups, which are limited primarily by the capacity of their members whose contributions are largely in addition to already full workloads. This further emphasizes the importance of the dedicated coordinator who can focus on implementing initiatives on behalf of the collective.

The evolution from mutually reinforcing activities to high-leverage activities highlights the potential for diverse approaches to coexist, each contributing to a shared goal. This evolution is evident in both the CSWB and TDAS. While both actively co-create new initiatives to achieve their goals, they also recognize and celebrate independent interventions that, even without direct coordination, align with and advance the shared objectives of their respective plans.

### Management → movement building

The Collective Impact model has proven valuable in providing the organizational infrastructure needed for cross-sectoral collaboration among local organizations on complex rural issues. Nevertheless, with both groups continuing to mature—although several years old, TDAS was formed during the COVID-19 pandemic, and CSWB formally launched in late 2023—the validity of critiques of this model’s inability to drive deep structural changes required to address health inequities has yet to be confirmed. Both initiatives have involved collective policy advocacy, in some cases beyond what individual organizations may have felt politically able to do (particularly TDAS, where the provincial government policies affecting substance use health frequently contradict best available evidence). Tensions between institutional roles and the political contexts in which they operate can limit their capacity for transformative change (Raphael, 2014). This highlights the need for complementary efforts, potentially led by other groups, to tackle root causes more comprehensively.

The World Health Organization’s framework for addressing social determinants of health emphasizes the importance of community-level action to reduce the vulnerabilities of disadvantaged people (Solar & Irwin,^2010^). TDAS and the CSWB Plan exemplify this meso-level action, also using multi-level governance mechanisms to inform higher levels of government about local priorities to create sustainable change.

## Conclusion

The need is clear for innovative, place-based, intersectoral approaches that address complex social problems, and that are compatible with, and reflective of, the realities of rural communities. Initiatives like the Ontario CSWB Plan and local drug and alcohol strategies address priorities held at local, provincial, and federal levels and lead to initiatives that would not have happened otherwise. These initiatives facilitate locally tailored interventions that balance local and regional collective implementation, working with the strengths of this rural region and avoiding over-burdening any single municipality.

As the highlighted initiatives continue to evolve, their success is hindered by inadequate support from existing funding structures and institutional limitations, and challenges with access to timely and meaningful health outcome data. Local public health can play a key role in facilitating this collaboration, meeting its organizational mandate while catalyzing action across sectors and disciplines and facilitating community engagement, but additional financial support for human resources and localized data collection are required to enable full implementation of the model. Complementing this work with additional advocacy to address root causes of inequity, and community engagement, can help ensure local rural communities thrive.

## Implications for policy and practice

What are the innovations in this policy or program?

- Public health-led regional implementation of the Collective Impact framework in rural Northern Ontario, Canada, with the local public health unit acting as the backbone agency, is an innovative way for local public health to meet its mandate while acknowledging the assets and challenges of rural settings.

What are the burning research questions for this innovation?

- What are critical factors for rural community collaboration on complex social issues?
- How can rural institutional actors effectively engage with communities that are sparsely populated and face barriers to transportation and Internet access?

## Footnotes

1 As of January 1, 2025, Timiskaming Health Unit has merged with another organization to form Northeastern Public Health (NEPH). For the purposes of this paper, we continue to use Timiskaming Health Unit as the organization that led the initiatives in the case study.
